# Interest of the BLAST paradigm and salivary markers for the evaluation of sleepiness in drivers

**DOI:** 10.3389/fnins.2022.991528

**Published:** 2022-09-07

**Authors:** Marine Thieux, Aurore Guyon, Vania Herbillon, Lydie Merle, Jean-Philippe Lachaux, Sabine Plancoulaine, Laurent Seugnet, Patricia Franco

**Affiliations:** ^1^Centre de Recherche en Neurosciences de Lyon (CRNL), Institut National de la Santé et de la Recherche Médicale (INSERM), Lyon, France; ^2^Centre de Référence Maladies Rares (CRMR) Narcolepsie-Hypersomnies Rares, Hôpital Femme-Mère-Enfant, Hospices Civils de Lyon (HCL), Lyon, France; ^3^CRESS, Inserm, INRAE, Université Paris Cité, Paris, France

**Keywords:** attention, vigilance, sleep, saliva, α-amylase, oxalate, biomarkers

## Abstract

**Objectives:**

Sleepiness is associated with decreased cognitive abilities and remains one of the main causes of fatal road accidents. The tools currently available to assess sleepiness, such as questionnaires, are subject to intra- and inter-individual variability, while multiple sleep latency tests are only feasible in few sleep laboratories. The main objective of this study was to explore new potential markers (neurocognitive, biological) to objectively assess sleepiness in drivers.

**Methods:**

A total of 186 drivers (median age 44 years, range 20–74 years, 73% men, 14% obese) were included during a break at a highway service area, in the morning, while on the road for vacation. Questionnaires on sleepiness and sleep characteristics (habitual and on the night before travel), the Bron-Lyon Attention Stability Test (BLAST), and two salivary samples (α-amylase and oxalate) were collected. Associations between measures of sleepiness [Epworth Sleepiness Scale (ESS), and Stanford Sleepiness Scale (SSS)], sleep characteristics, neurocognitive, and biological markers were tested using regression models adjusted for confounding factors.

**Results:**

The night before travel, 83% of the drivers reduced their sleep time and 30% slept 5 h or less. The higher the number of miles to be traveled, the higher the decrease, and the shorter the sleep time. The night before travel, 18 and 24% of the drivers complained of poor sleep quality and difficulty falling asleep. The sleep characteristics on the night before travel were associated with the habitual sleep characteristics. At the time of the test, 47% of the drivers scored pathologically on the SSS. Poor sleep quality and difficulty falling asleep the night before travel were associated with increased sleepiness as assessed by the SSS and decreased attentional ability as assessed by the BLAST. No association between salivary markers and acute sleepiness was observed.

**Conclusions:**

The sleep characteristics of the night before travel were associated with sleepiness and attentional performance. The SSS and the BLAST could be used by individual drivers in a self-evaluation context. Biological markers showed a high variability and limited association with sleep parameters across subjects, emphasizing the need for within-subject designs to assess their usefulness.

## Introduction

One in five French person has an abnormal tendency to fall asleep or experiments sleepiness during the day (Léger et al., 2012). Among them, 11% report daily sleepiness and 7% report not being able to resist the urge to fall asleep during the day, at least three times a week. Sleepiness is not always perceived (Van Dongen et al., 2004; Schreier et al., 2015) and can even occur with a subjective feeling of vigilance. Sleepiness can result from sleep perturbations (e.g., poor sleep habits, shift work, sleep disordered breathing) and can be augmented by organic pathologies (e.g., obesity, chronic obstructive pulmonary disease, cardiovascular pathology). Today, sleep deprivation is commonplace, with major implications for the health of individuals (e.g., increased risks of metabolic, cardiovascular, psychiatric disorders) (Garbarino et al., 2016). While it is a very common phenomenon, sleepiness is also the leading cause of fatal road accidents (Léger and Ement, 2015). Even if sleep deprivation occurs only the night before travel, it can have a negative impact on driving ability the next day (Maia et al., 2013) as sleep deprivation is related to decreased visual, cognitive, and motor alertness (Connor, 2002). This decrease is similar to that induced by alcohol consumption (Powell and Chau, 2011). Importantly, sleep deprivation is common before leaving on vacation: 50% of drivers reduce their sleep time the night before departure, and the more miles to drive, the greater the decrease (Philip et al., 1997). Sleep hygiene and sleepiness management thus appear as major strategies to prevent road accidents.

Unlike what is available for alcohol consumption or speeding, there is currently no optimal tool to assess sleepiness in drivers (Pajcin et al., 2019a). Although subjective techniques, such as scales and questionnaires, are attractive due to their ease of use and low cost, they are sensitive to inter- and intra-individual variability. They also do not represent reliable diagnostic tools as it has been shown that it is particularly difficult to correctly perceive and self-report sleepiness (Van Dongen et al., 2004; Schreier et al., 2015; Dauvilliers et al., 2017; Peter-Derex et al., 2020; Des Champs de Boishebert et al., 2021). Conversely, objective techniques such as the Multiple Sleep Latency Test (MSLT) or Maintenance of Wakefulness Test (MWT), which can only be performed in few sleep laboratories, are time-consuming, far from real-life conditions, and do not correlate with subjective measures (Wise, 2006; Peter-Derex et al., 2020). Moreover, while these different measures are sensitive to sleep propensity, they do not detect the rapid physiological changes (micro or local sleeps) associated with sleepiness and which can lead to brief attentional lapses under certain conditions, increasing performance instability (Doran et al., 2001; Peiris et al., 2006; Lim and Dinges, 2008; Andrillon et al., 2019, 2021; Des Champs de Boishebert et al., 2021). Other measures, such as the Psychomotor Vigilance Test (PVT), can assess the quality of vigilance and are sensitive to microsleeps (Lim and Dinges, 2008) but only provide an overall estimate of attentional performance, falling to capture the moment-to-moment attentional state (Petton et al., 2019; Thieux et al., 2019). The evaluation of the latter is particularly relevant as only a few seconds of sleepiness, and thus inattention, can be fatal on the road.

An easy-to-use attention test that could assess the micro-fluctuations in vigilance repeatedly during travel would therefore be of great value. The Bron-Lyon Attention Stability Test (BLAST), which measures variability on a second-by-second basis, can assess brief fluctuations of attention, and could thus be useful to assess the stability and efficiency of performance related to sleepiness in drivers (Petton et al., 2019; Thieux et al., 2019). In addition, salivary markers, which are easily accessible and can be collected in a non-invasive manner, could also be used to assess sleepiness. Salivary α-amylase (sAA) is an enzyme produced by the salivary glands (i.e., particularly the parotid glands) innervated by the autonomic nervous system, especially the sympathetic nervous system (SNS), involving noradrenaline (NA) (Rohleder and Nater, 2009; Faraut et al., 2020). NA is also implicated in the regulation of wakefulness and vigilance (Pajcin et al., 2019b), making sAA an indirect measure of noradrenergic activity, and therefore of vigilance (Rohleder and Nater, 2009; Pajcin et al., 2017, 2019b). The expression of salivary α-amylase (sAA) has been shown to be related to sleep debt in animals and humans (Seugnet et al., 2006; Bachmann et al., 2012), although the results in humans are still contradictory regarding the exact association between sAA and sleep deprivation. In healthy subjects, decreased sAA levels were reported after sleep restriction or deprivation and were associated with diminished cognitive performances (Rabat et al., 2016; Pajcin et al., 2017, 2019a; Faraut et al., 2020). In contrast, increased sAA levels were reported with sleep debt (Nater et al., 2007; Figueiro and Rea, 2011; Bachmann et al., 2012; O'Leary et al., 2015; Van Lenten and Doane, 2016; Unno et al., 2017). Oxalate, on the other hand, was identified in a metabolomic study in sleep restricted rats and humans, and shown to be downregulated by sleep loss (Weljie et al., 2015). Thus, sAA and oxalate could be considered as biomarkers of sleep pressure. Although these markers may constitute innovative tools for the objective assessment of sleepiness, large-scale studies in real-life situations are needed to confirm their interest.

The main objective of this study was to explore new potential neurocognitive and biological markers to assess sleepiness in drivers. It was hypothesized that these markers would be modified according to sleep characteristics and sleepiness levels.

## Materials and methods

### Participants

During 2 study sessions on June 30 and October 20, 2018, drivers were included during a break at 2 different highway service areas (Montélimar and Saint-Rambert d'Albon, France) while on the road for vacation. They were at least 20 years old and had their driving license at least 2 years prior to their inclusion. They had not undergone a trans-meridian travel within the last month nor had eaten, drunk, or chewed gum in the past 30 min. They had no self-reported diagnosed medical disorders (e.g., central hypersomnolence, cancer, diabetes, cardiovascular or thyroid disease, chronic pain, autoimmune, neurodegenerative or mood disorders) or treatment (e.g., antihistaminic) that could impact vigilance levels at the time of the study.

### Procedure

**Figure 1:**
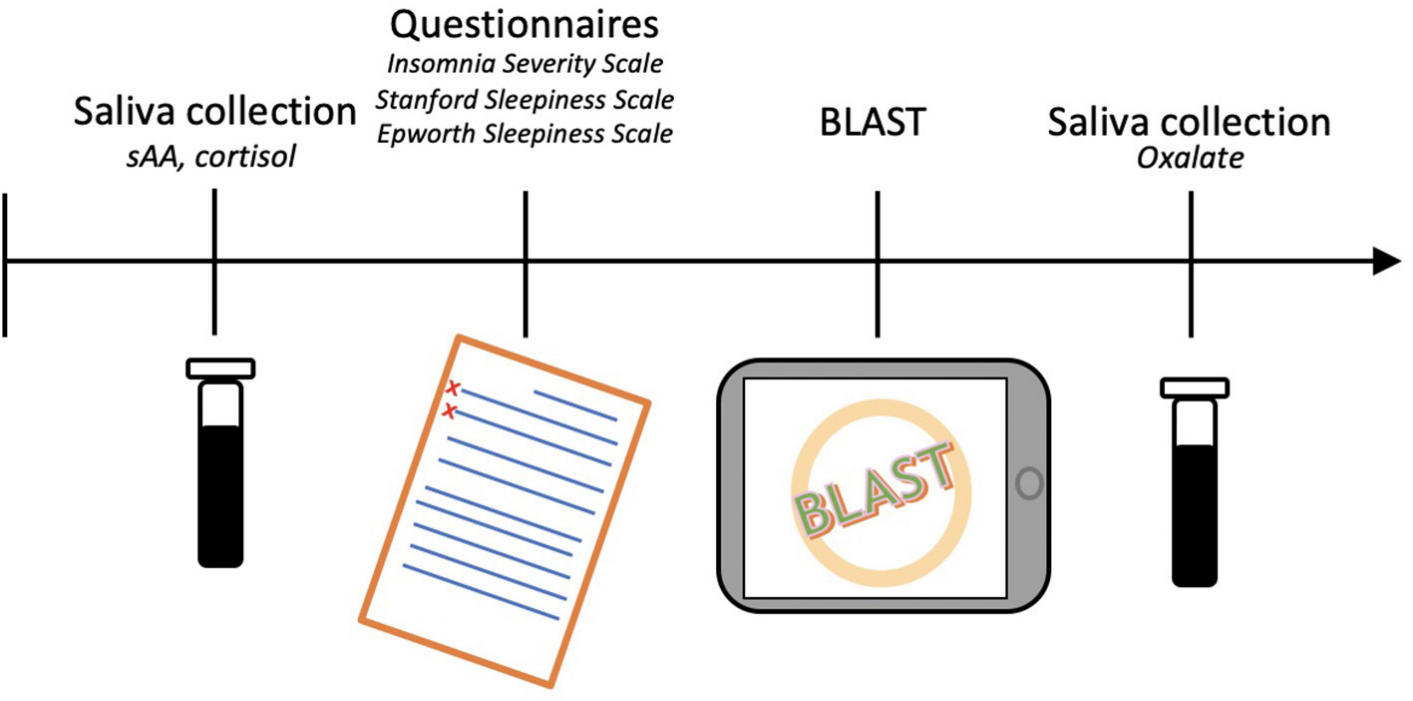
Study procedure for each participant.

For each driver, the study began with the collection of an initial saliva sample. Next, questionnaires were filled in and the BLAST was performed (Petton et al., 2019). Finally, a second saliva sample was collected. The total procedure lasted approximately 20 min (Figure 1). Drivers were compensated with a coffee token and a bag containing gifts (neck pillow, earplugs, and sleep mask).

#### Saliva collection

Participants were asked to passively hold a piece of saliva-absorbing cotton in their mouth for one min. The first sample collected sAA and cortisol, whereas the second sample collected oxalate. For the second collection, 1 mL of RNA later ^TM^ was added directly to the soaked cotton to stabilize the sample. The samples were immediately frozen, stored on dry ice, then entrusted to the transporter for transfer to the laboratories that performed the analyses. The assays were performed for sAA, oxalate, and cortisol. Cortisol levels were assessed to control for the potential effect of stress on sAA (Chatterton et al., 1996; Nater et al., 2007; Bright et al., 2014). No collection of the biological samples was performed. A logarithmic transformation was applied on sAA and cortisol levels while a square-root transformation was applied on oxalate levels to account for positively skewed distributions (Supplementary Figure S1) (Granger et al., 2007; Nater et al., 2007; Rohleder and Nater, 2009; Van Lenten and Doane, 2016; Pajcin et al., 2019a).

#### Questionnaires

Demographic characteristics were collected before starting the questionnaires: age (in years), sex, height (cm), weight (kg), smoking (yes/no), night work (yes/no), and socioeconomic level (SEL, according to the French National Institute of Statistics and Economic Studies classification). Participants were then asked about their sleep habits: total sleep time (TST), time of sleep onset and waking-up, snoring (yes/no). The sleep characteristics of the night before travel were also collected: TST, time of sleep onset and waking-up, sleep quality (good/bad), difficulty falling asleep (yes/no), and sleep latency greater than 30 min (yes/no). Participants were also asked about potential sleepiness experiences on the road within the last year: sleepiness on the road (yes/no), stops due to sleepiness (yes/no). Their management of rest during the travel was also collected: total number of miles to drive, number of miles already driven, and naps (yes/no). Finally, the participants filled in 3 questionnaires: (a) *The Epworth Sleepiness Scale* (ESS) which assesses long-term sleepiness (past months) by evaluating the likelihood of falling asleep in 8 daily-life situations estimated on a 4-point Likert scale. The total score is the sum of the scores of the 8 items, a higher score representing greater sleepiness and the pathological threshold being higher than 10 (Johns, 1991); (b) *The Stanford Sleepiness Scale* (SSS) which assesses the sleepiness at the time of the saliva collection. The subjective sleepiness level is quantified on a 7-point scale varying from “very alert” to “excessively sleepy”. A higher score represents a greater sleepiness and the pathological threshold is set to be higher than 2 (Hoddes et al., 1973; Herscovitch and Broughton, 1981); and (c) *The Insomnia Severity Index (ISI)* which assesses insomnia severity *via* 7 items scored on a 5-point Likert scale. The higher the total score, the more severe the symptoms. The pathological threshold is set to be higher than 10 (Bastien et al., 2001; Morin et al., 2011).

#### BLAST

**Figure 2:**
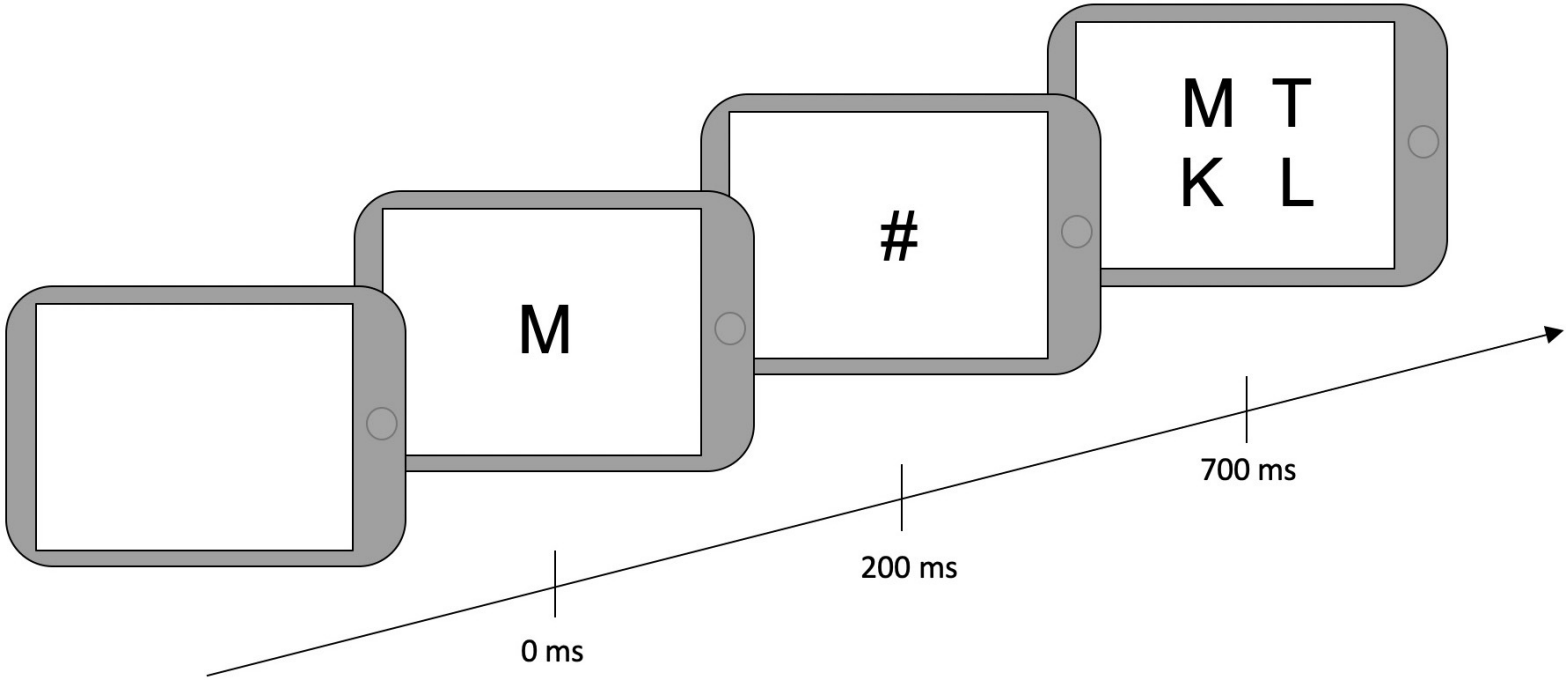
Typical trial of the BLAST paradigm.

After filling out the questionnaires, drivers were told that they would play a game that aimed to find a balance between speed and accuracy. They were instructed to find a target letter in an array of four letters. First, the target letter appeared on the screen for 200 milliseconds (ms), followed by a mask (#) for 500 ms, followed by a 2-by-2 array of four letters (Figure 2). They were asked to provide responses manually on the tablet. “Yes” responses were given when one of the letters was the same as the target, using the non-dominant hand. “No” responses were provided when the target letter was not presented in the array, using the dominant hand. The next trial began 800 ms after array presentation if a response was given and 3800 ms after array presentation if no response was given. The total testing time was 2 min and 30 sec; the number of trials was not predefined.

**Figure 3:**
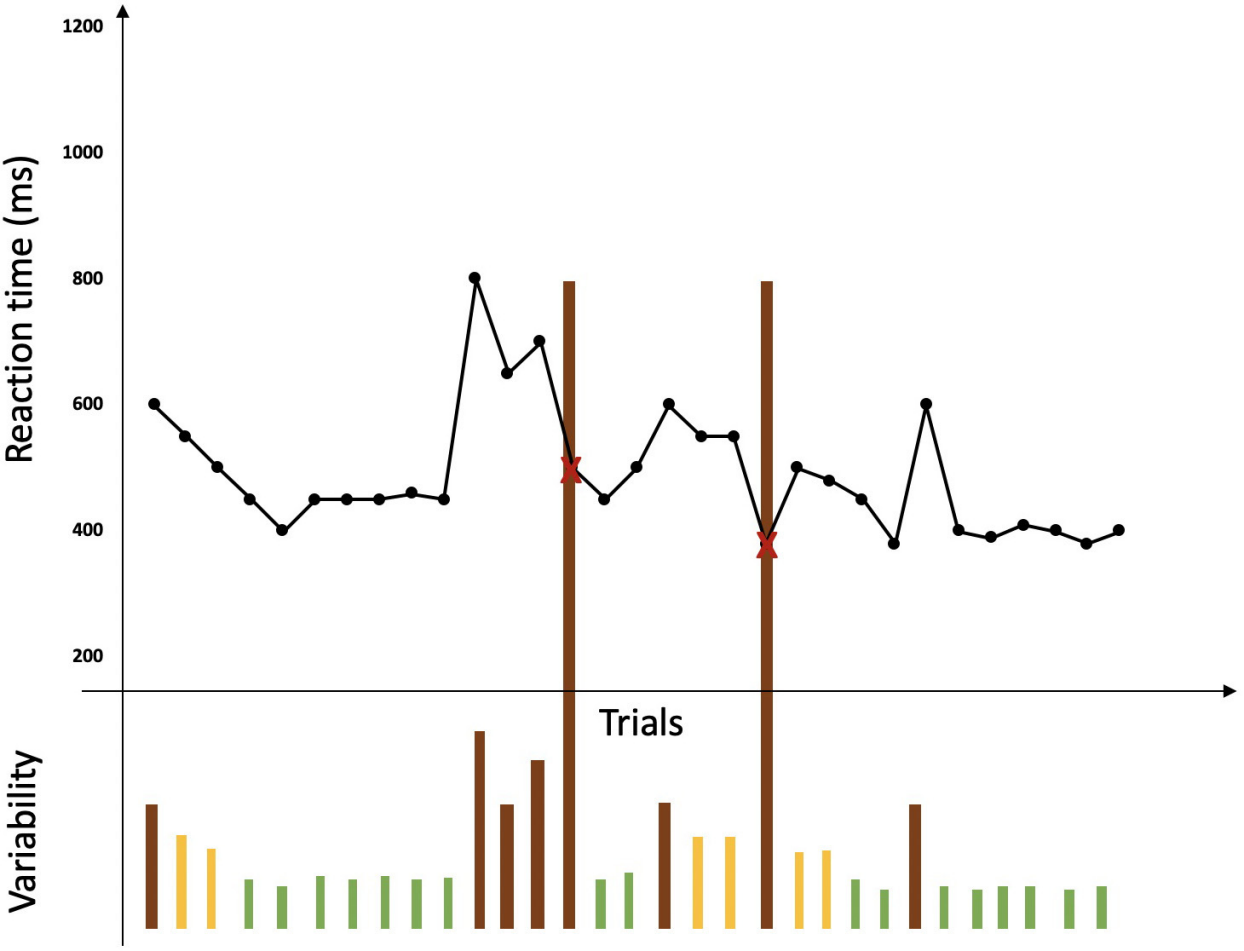
Representation of a BLAST session adapted from Petton et al. (2019). Reaction times for each trial are represented by a black point and errors are represented by a red cross. No lack of response is represented here. Variability in performance is represented under the curve of reaction times by bars: green bars represent a good stability of performance while orange bars represent instability and brown bars represent lapses associated with errors or a large variability in reaction time.

Two specific behavioral measures of the BLAST were selected: BLAST-stability and BLAST-intensity, which were created to capture number, duration, and localization of Momentary Lapses of Attention (MLA) during the task (Petton et al., 2019) (Figure 3). BLAST-stability reveals the ability to produce long series of correct responses with a stable reaction time, independently of speed. BLAST-intensity reveals the ability to produce long series of fast and correct responses. For both measures, the higher the score, the better the attention [for a more detailed description of these measures and of the psychometric properties of the BLAST, see (Petton et al., 2019; Thieux et al., 2019)].

### Statistical analysis

Statistical analyses were conducted using R software (R Core Team, 2020). Continuous measures were expressed as median and range; dichotomous and polytomous measures were expressed as *n* and percentage. Subjects with random BLAST performances (i.e., > 50% errors), with missing data for saliva levels or time of collection were excluded from the analysis.

The variables to explain were the new potential values of sleepiness: sAA and oxalate levels, BLAST-stability, and BLAST-intensity. Sleepiness measures (ESS total score and SSS total score) were used as explanatory variables. The co-variables were all the other collected values. Directed acyclic graphics (Textor et al., 2016) were produced to better visualize the necessary adjustments for estimating the effect of sleepiness on the variables to explain (Supplementary Figure S2).

First, using non-parametric tests, bivariate analyses were conducted to analyze the association between variables to explain, sleepiness measures, and each co-variable. Then, correlations and associations were analyzed between variables with significant effect in the bivariate analyses. Finally, multivariate models with each significant co-variable adjusted for the time of inclusion or total awake time were computed. To assess factors influencing the new markers, generalized linear models were applied. Logarithmic models were also computed on sAA and oxalate divided into two categories (i.e., high and low) according to a median split. The choice of the best model was made according to the Akaike information criterion and Likelihood values. A false discovery rate correction for multiple comparisons was used. The statistical significance value was set to *p* < 0.05.

## Results

### Descriptive analysis

A total of 186 drivers were included in the cohort. Their median age was 44 years, 73% were men, and 14% were obese. Among all drivers, 22% were smokers (80% of whom were men), and 17% worked nights (84% of whom were men). In daily life, the median TST was 7:00 h, 51% slept 7:00 h or less, and 16% slept 6:00 h or less. Snoring was reported by 43% of the drivers (87% of whom were men) and 21% reported insomnia (ISI >10, 67% were men; Tables 1, 2). In the past year, 49% reported at least one episode of sleepiness on the road, and 37% reported having stopped while driving due to sleepiness (Table 1). The ESS was pathological in 17% of the drivers (Table 2).

**Table 1:** Demographic, habitual sleep, and pre-travel sleep characteristics.

**Demographic characteristics**	**Median (range)**	**N**
Age	44 (20–74)	186
Sex, men, % (N)	73% (135)	186
Body Mass Index (BMI)	25 (17–49)	184
Obesity, % (N)	14% (26)	184
Smokers, % (N)	22% (41)	186
Night work, % (N)	17% (31)	182
Socio-economic level, % (N)		184
*Farmers*	2% (3)	
*Artisans, shopkeepers and chief executive officers*	4% (8)	
*Executive and intellectual professions*	32% (58)	
*Intermediate professions*	16% (30)	
*Employees*	23% (42)	
*Workers*	11% (21)	
*Students, retired, and unemployed*	12% (22)	
**Habitual sleep and sleepiness characteristics**		
TST, hours	7 (4.5–10)	155
Time of sleep onset	11:00 pm (8:20 p.m.−4:00 a.m.)	154
Time of waking-up	7:15 am (4:00 a.m. – 11:00 a.m.)	155
Snoring, % (N)	43% (79)	183
Sleepiness on the road, % (N)	49% (91)	185
Stops while driving due to sleepiness, % (N)	37% (68)	184
**Pre-travel sleep and sleepiness characteristics**		
TST, hours	6 (1.5-11.5)	186
Time of sleep onset	1:00 am (9:00 p.m.−6:00 a.m.)	186
Time of waking-up	6:00 am (7:30 p.m.−11:30 a.m.)	186
Sleep quality, % (N)		185
*Good*	82% (152)	
*Bad*	18% (33)	
Difficulty falling asleep, % (N)	24% (45)	185
Sleep latency > 30 min, % (N)	18% (32)	177
Total awake time after wake up, hours	5.3 (1–12.5)	186
Number of miles traveled	186 (6–528)	184
Naps, % (N)	12% (21)	182

Values are reported as median (range) unless otherwise specified. BMI, Body Mass Index; TST, Total Sleep Time.

**Table 2:** Questionnaires and BLAST characteristics.

**Questionnaires**	**Median (range)**	**N**
Stanford Sleepiness Scale		182
SSS, total score	2 (1–6)	
SSS, pathological, % (N)	47% (85)	
Epworth Sleepiness Scale		183
ESS, total score	6 (0–17)	
ESS, pathological, % (N)	17% (31)	
Insomnia Severity Index		140
ISI, total score	6 (0–21)	
ISI, pathological, % (N)	21% (30)	
**BLAST**		186
BLAST-stability	28 (0–61)	
BLAST-intensity	54 (3–81)	

Values are reported as median (range) unless otherwise specified. SSS, Stanford Sleepiness Scale; ESS, Epworth Sleepiness Scale.

**Figure 4:**
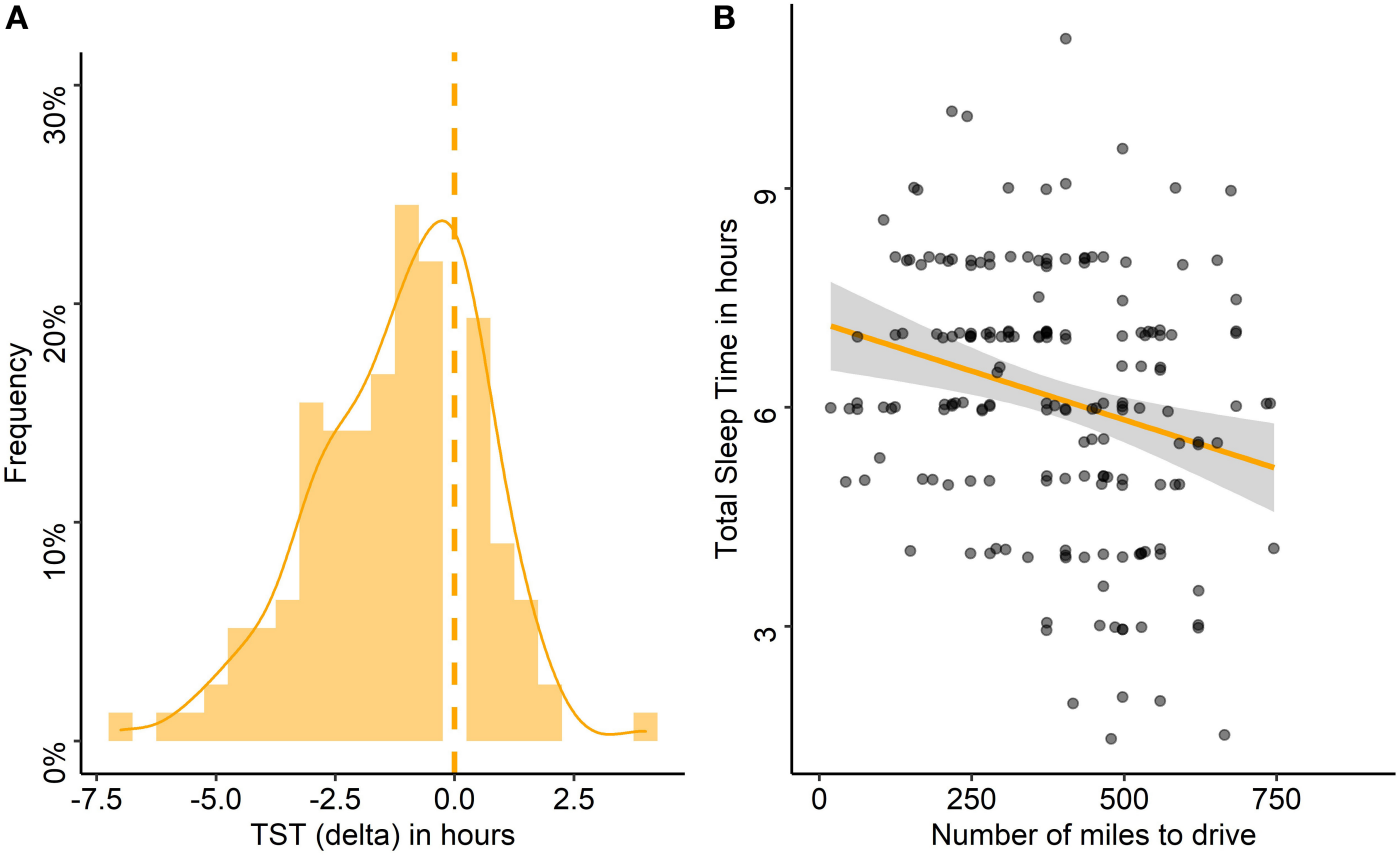
Differences between habitual and pre-travel total sleep time and association with the number of miles to drive. **(A)** Frequency of delta between habitual total sleep time (TST) and TST on the night before travel in the entire cohort, the dashed line stands for the absence of difference. **(B)** Association between TST the night before travel and number of miles to drive. Each point represents the TST of one participant according to the number of miles to drive.

The night before travel, the median TST was 6:00 h and 30% slept 5:00 h or less (Table 1). A decrease in TST the night before travel compared with the habitual TST was found in 83% of the drivers, with a median (range) decrease of 1 h (−7–+4 h; Figure 4A). Poor sleep quality was reported by 18% of the drivers, and 24% reported having had difficulty falling asleep (18% of whom declared a sleep latency > 30 min; Table 1).

The median (range) time of inclusion was 11:15 am (7:45 a.m.−2:40 p.m.). At the time of the test, drivers had been awake for a median (range) of 5.3 h (1–12.5 h), 12% had napped, and they had driven a median (range) of 186 miles (6–528 miles; Table 1). The SSS was pathological in 47%. Sleepiness score, BLAST-stability, and BLAST-intensity scores are reported in Table 2. A high number of drivers performed below normative values on the BLAST. Saliva concentrations for the entire sample and according to sex are reported in Table 3 (for saliva concentrations according to other factors, see Supplementary Table S1).

**Table 3:** Levels of saliva markers according to sex.

	**Men**	**N**	**Women**	**N**	**Total**	**N**
α-amylase, U/ml, mean (SD) median (range)	73.12 (80.38) 41.23 (2.49–482)	135	76.01 (72.43) 57.83 (3.67–360.20)	51	73.91 (78.10) 50.09 (2.49–482)	186
Oxalate, U/ml, mean (SD) median (range)	0.028 (0.025) 0.020 (0.002–0.112)	135	0.038 (0.029) 0.031 (0.001–0.197)	51	0.035 (0.028) 0.029 (0.001–0.197)	186
Cortisol, μg/dL, mean (SD) median (range)	0.224 (0.145) 0.193 (0.031–0.893)	135	0.212 (0.105) 0.178 (0.063–0.575)	51	0.221 (0.135) 0.19 (0.03–0.89)	186

### Associations

#### Associations between co-variables

There were significant associations between co-variables (Supplementary Figure S3).

Stopping while driving due to sleepiness in the past year was more common among those who reported sleepiness on the road or snoring than in drivers without sleepiness (91 vs. 1%, *p* < 0.001) or snoring (59 vs. 41%, *p* < 0.01). Men snore more than women (53 vs. 18%, *p* < 0.001), and men or drivers who snore had a higher BMI than women (median BMI: 25.7 vs. 22.1, *p* < 0.001) or drivers who do not snore (median BMI: 26.3 vs. 24, *p* < 0.001).

The sleep characteristics of the night before travel were associated with the habitual sleep characteristics. The pre-travel TST was associated with habitual TST (rho = 0.31, *p* < 0.001), and both were significantly higher in women than in men (median TST: 7 h vs. 6 h, *p* = 0.02 and 7 h 45 vs. 7 h, *p* = 0.03, respectively). The time of sleep onset the night before travel was associated with habitual time of sleep onset (rho = −0.46, *p* < 0.001) as was the time of waking up (rho = 0.24, *p* = 0.01). The night before travel, drivers with poor sleep quality, difficulty falling asleep, or sleep latency >30 min had higher ISI scores than drivers with good sleep quality (median ISI scores: 9 vs. 5, *p* < 0.001), no difficulty falling asleep (median ISI scores: 9 vs. 5, *p* < 0.001), or shorter sleep latency (median ISI scores: 9.5 vs. 5, *p* < 0.001). Poor sleep quality, difficulty falling asleep, and sleep latency >30 min the night before travel were associated with each other (*p* < 0.001 for all the associations). Among drivers with poor sleep quality the night before travel, 63% also reported difficulties falling asleep and 45% a sleep latency > 30 min (compared to 16 and 12% in drivers with a good sleep quality, respectively). Among those with difficulties falling asleep, 64% reported a sleep latency > 30 min (vs. less than 1% in drivers without difficulties).

The sleep characteristics of the night before travel were associated with the travel characteristics. TST as well as the time of sleep onset and waking up were associated with the number of miles to drive (Figure 4B), the time of departure and thus, the number of miles already traveled, the time of inclusion, and the total time awake since wake up (Table 4). The more miles to drive, the earlier the time of departure (rho = −0.52, *p* < 0.001). The number of miles to drive as well as the number of miles already traveled were lower for women than for men (median: 304 vs. 404 and 149 vs. 227 miles, respectively, *p* = 0.03 for both), and the departure time was later for women than for men (median: 8 a.m. vs. 6 a.m., *p* = 0.03). Drivers who napped had more miles to travel (median: 559 vs. 373 miles, *p* < 0.001), had already driven more miles (median: 373 vs. 186 miles, *p* < 0.001), and had started the trip earlier (median: 4 a.m. vs. 7 a.m., *p* = 0.01); they also reported more frequent sleepiness-related driving stops in the past year (67 vs. 32%, *p* = 0.03) than those who did not nap.

**Table 4:** Correlations between pre-travel sleep characteristics and travel characteristics.

	**TST**	**Time of sleep onset**	**Time of waking-up**
Number of miles to drive	rho = −0.24 *p* = 0.003	rho = 0.18 *p* = 0.04	rho = −0.43 *p* < 0.001
Departure time	rho = 0.49 *p* < 0.001	rho = −0.33 *p* < 0.001	rho = 0.75 *p* < 0.001
Number of miles traveled	rho = −0.28 *p* < 0.001	rho = 0.21 *p* = 0.01	rho = −0.49 *p* < 0.001
Time of inclusion	rho = 0.23 *p* = 0.01	rho = −0.34 *p* < 0.001	rho = 0.47 *p* < 0.001
Total time awake since wake-up	rho = −0.48 *p* < 0.001	rho = 0.18 *p* = 0.03	rho = −0.63 *p* < 0.001

The delta between habitual and pre-travel TST was associated with the number of miles to drive (rho = −0.20, *p* = 0.03), the time of waking up and of departure (rho = 0.59, *p* < 0.001; rho = 0.40, *p* < 0.001, respectively) and thus, with the number of miles already traveled (rho = −0.25, *p* = 0.01) and the total awake time (rho = −0.48, *p* < 0.001). The larger the delta between habitual and pre-travel TST, the lower the pre-travel TST, the higher the number of miles to drive, the earlier the wake-up and departure time, and thus the higher the number of miles already traveled and the total awake time. This delta in TST as well as the time of waking up on the day of departure were associated with cortisol levels (rho = −0.19, *p* = 0.04 and rho = −0.23, *p* = 0.01, respectively), which were also associated with the time of departure (rho = −0.22, *p* = 0.02) and time of inclusion (rho = −0.37, *p* < 0.001). The greater the reduction of pre-travel compared to habitual TST and the earlier the wake-up time, the higher the cortisol levels. Thus, the earlier the departure and time of inclusion, the higher the cortisol levels.

#### Factors influencing chronic sleepiness

Smokers had higher levels of chronic sleepiness than non-smokers (median: 8 vs. 6, *p* = 0.04). Compared to those with ISI < 10, drivers with a pathological ISI score had a higher ESS score (median: 5 vs. 8, *p* = 0.04). The higher the ISI, the higher the ESS (rho = 0.26, *p* = 0.04). Drivers who had difficulty falling asleep or poor sleep quality had higher ESS scores than those who had no difficulty (median: 8 vs. 6, *p* = 0.04) or good sleep quality (median: 8 vs. 6, *p* = 0.04).

#### Factors influencing acute sleepiness

**Figure 5:**
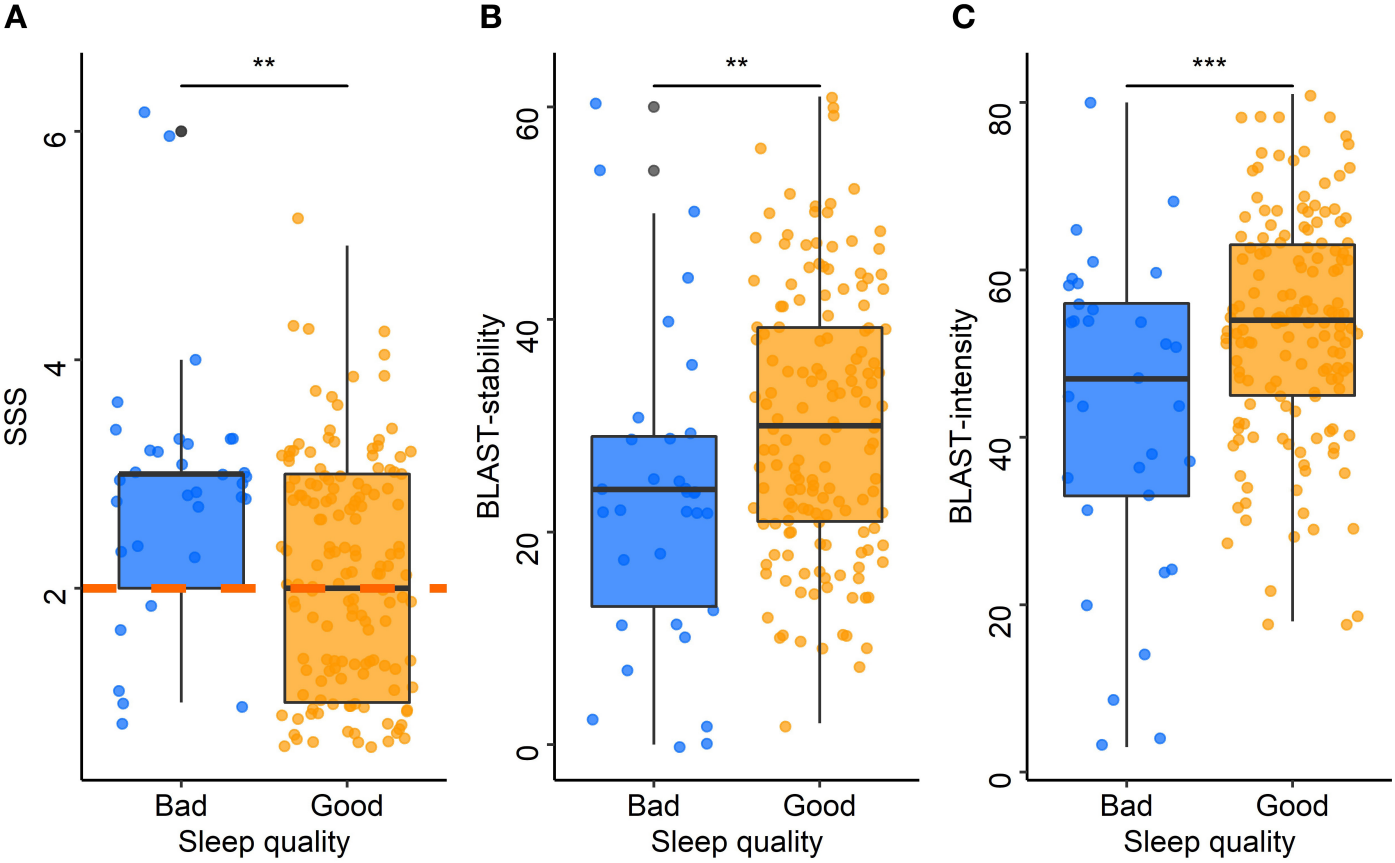
Acute sleepiness, BLAST-stability, and BLAST-intensity according to sleep quality. Each point represents the median Stanford Sleepiness Scale score **(A)**, BLAST-stability **(B)**, and BLAST-intensity **(C)** for each driver according to the sleep quality the night before travel (blue: bad, orange: good). The central line of boxplots corresponds to the median of each score, the upper and lower parts correspond to the first and third quartiles. The orange dashed line **(A)** represents the pathological threshold of the SSS. Difference between groups is represented by stars: ***: *p* ≤ 0.001; **: *p* ≤ 0.01.

ESS scores were positively correlated with SSS scores (rho = 0.23, *p* = 0.01): the higher the chronic sleepiness, the higher the sleepiness at the time of the test. Over the past year, drivers who experienced sleepiness on the road or had to stop because of sleepiness had higher SSS scores than those who did not (median: 3 vs. 2 for both, *p* < 0.001 and *p* = 0.01, respectively). SSS scores were positively correlated with the ISI (rho = 0.39, *p* < 0.001). The SSS was also associated with the sleep characteristics of the night before travel. Drivers with poor sleep quality (Figure 5A) or difficulty falling asleep had higher SSS scores than those with good sleep quality (median: 3 vs. 2, *p* = 0.01) or no difficulty falling asleep (median: 3 vs. 2, *p* = 0.01). Among drivers with poor sleep quality, 73% had a pathological SSS, and among those with difficulty falling asleep, 64% had a pathological SSS. Compared to those who did not nap, SSS was higher among drivers who had napped during the trip (median: 3 vs. 2, *p* = 0.02).

### New potential markers of sleepiness

#### Neurocognitive markers

The models adjusted for the time of inclusion and for the total awake time showed that BLAST-stability was significantly associated with sleep quality (*p* = 0.01, for both models; Figure 5B) and sleep latency (*p* = 0.04, for both models). The models adjusted for the time of inclusion and for the total awake time showed that BLAST-intensity was associated with sleep quality (*p* < 0.001, for both models; Figure 5C). Drivers with a poor sleep quality or a sleep latency > 30 min had lower attention scores than those with a good sleep quality (median BLAST-stability: 24 vs. 30; median BLAST-intensity: 47 vs. 54) and a sleep latency < 30 min quality (median BLAST-stability: 23 vs. 30; median BLAST-intensity: 52 vs. 54) on the night before travel. There was no significant association between BLAST scores and sleepiness scales (ESS, SSS) or between BLAST scores and other co-variables.

#### Biological markers

##### Salivary α-amylase

The adjusted models run with log-transformed sAA concentrations showed no significant association between sAA levels, sleepiness scores, or other co-variables.

The adjusted models run with median split sAA levels showed that sex (*p* = 0.03 for the adjusted model on the time of inclusion and *p* = 0.05 for total awake time), snoring (*p* = 0.03 for the adjusted model on the time of inclusion and *p* = 0.05 for the total awake time), and pre-travel sleep latency (*p* = 0.03 for the adjusted model on the time of inclusion and *p* = 0.02 for the total awake time) were independently associated with sAA concentrations. Low sAA concentrations were more common in men than in women (54 vs. 38%), in people who snore compared to those who do not (57 vs. 42%), and in drivers with a sleep latency <30 min compared to those with a sleep latency >30 min (54 vs. 31%). In multivariate analyses, these independent effects remained significant. There was no significant association between sAA concentrations and sleepiness scales (ESS, SSS), or between sAA concentrations, cortisol levels (Supplementary Figure S4), and other co-variables.

##### Salivary oxalate

**Figure 6:**
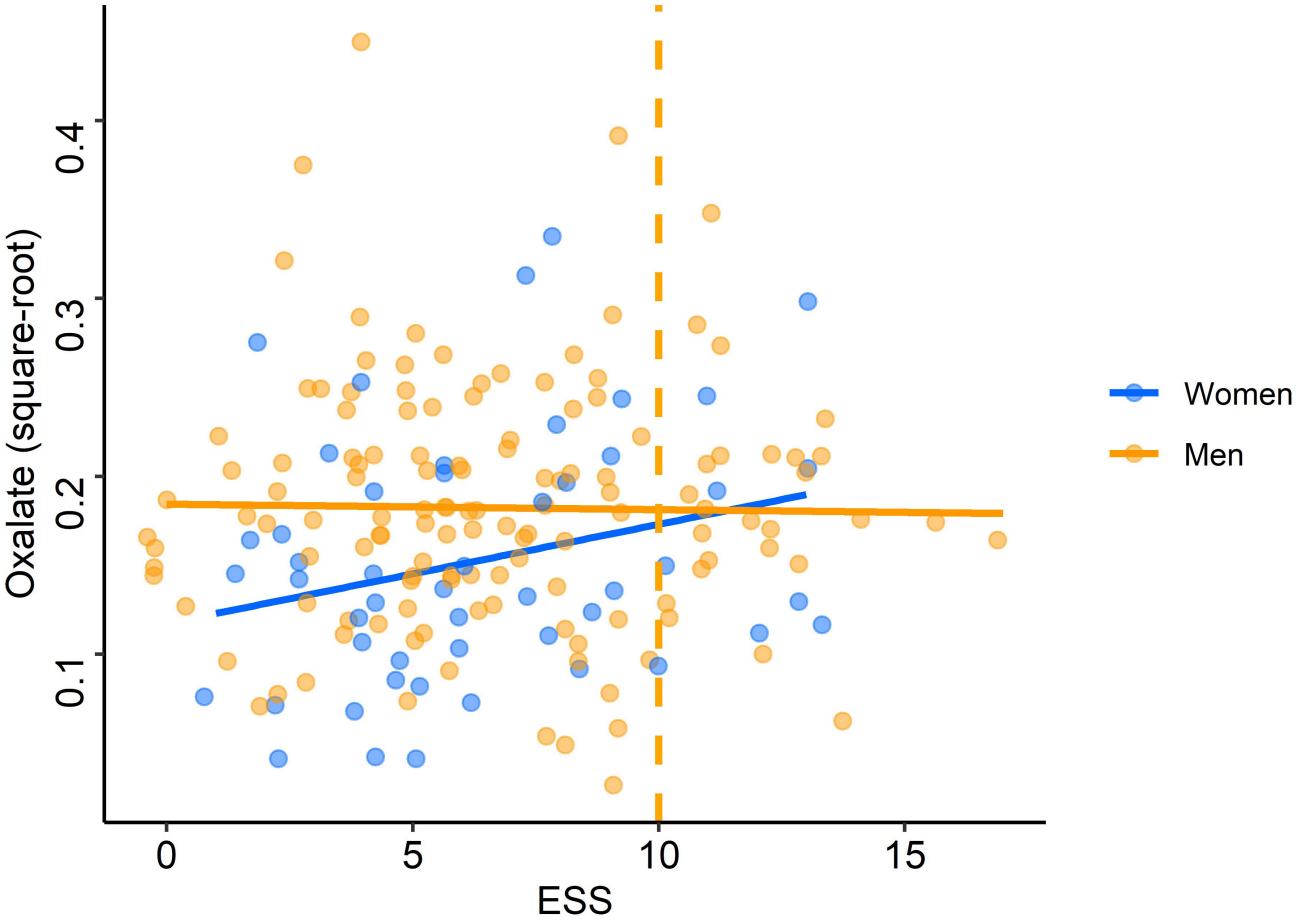
Oxalate levels according to Epworth Sleepiness Scale scores. Each point (blue: women, orange: men) represents oxalate concentration (after square-root transformation) for each driver according to the score on the Epworth Sleepiness Scale (ESS). The orange dashed line represents the pathological threshold of the ESS.

The adjusted models run on square-root transformed oxalate concentrations showed that oxalate concentrations were significantly associated with sex (*p* = 0.02 for the adjusted model on time of inclusion and *p* = 0.03 for the total awake time) and smoking (*p* < 0.001 for both adjusted models). Smokers and men had significantly higher oxalate levels than non-smokers (median: 0.040 vs. 0.026 U/ml) (Supplementary Table S1) and women (median: 0.031 vs. 0.020 U/ml), respectively (Table 3). These independent effects remained significant at the multivariate level. In multivariate analyses, there was an interaction between ESS and sex: when the ESS was low, men had higher oxalate levels than women but, when the ESS was high, women had higher oxalate levels than men (*p* = 0.02 for both adjusted models; Figure 6).

The adjusted models conducted on median split oxalate levels confirmed the effect of sex (*p* = 0.01 for both adjusted models) and smoking (*p* = 0.02 for both adjusted models) but not the interaction between ESS and sex (Supplementary Figure S5). Low oxalate levels were more commonly found in women than in men (67 vs. 44%) and in non-smokers compared to smokers (55 vs. 31%). These independent effects remained significant at the multivariate level. There was no other significant association between oxalate levels and sleepiness scales (ESS, SSS) or between oxalate levels and other co-variables.

## Discussion

The main findings highlight that, under ecological conditions, SSS and BLAST-scores reflect sleepiness and its attentional repercussions in drivers. However, salivary biomarkers showed a high variability, related to this real-life condition, which likely limited its association with sleep parameters.

In the past year, nearly half of the cohort reported at least one episode of sleepiness while driving and more than a third stopped while driving due to sleepiness, a result similar to that reported in the US population (Powell and Chau, 2011). Herein, the proportion of drivers with chronic sleepiness, as assessed by the ESS, was close to the 14–17% and 19% found in the French population (Meslier et al., 2007; Fuhrman et al., 2012). Drivers who experienced sleepiness while driving and those who usually snore were more likely to stop while driving due to sleepiness. Interestingly, the latter were predominantly men and / or people with a high BMI. Moreover, smokers also reported higher levels of sleepiness than non-smokers. This is noteworthy as it is well known that individuals who smoke, snore, or have a high BMI, particularly men, are at risk of sleep apnea syndrome (Wetter, 1994; Deegan and McNicholas, 1996). Chronic sleepiness may thus reflect the deleterious impact of sleep fragmentation on daytime functioning. Chronic sleepiness was also strongly associated with acute sleepiness: drivers who scored high on the ESS and drivers who experienced sleepiness on the road or had to stop while driving because of sleepiness in the past year had high SSS scores. A pathological threshold for acute sleepiness has been reported in nearly half of the population, highlighting the need for regular breaks while driving. A nap could also be recommended (Reyner and Horne, 1997; Philip et al., 2006; Sagaspe et al., 2007) but as previously reported, subjective sleepiness does not systematically improve after a nap (Lenné et al., 2004). In fact, the SSS herein was higher in drivers who took a nap during the trip. This may be understood as a confounding effect, because they also had more miles to travel, had already driven more miles, and started the trip earlier. However, this may also reflect persistent sleep inertia after a nap (Lenné et al., 2004) or a better awareness of sleepiness, as drivers who napped also made frequent stops because of sleepiness in the past year.

The sleep characteristics of the night before travel were strongly associated with habitual sleep characteristics. Those with usual sleep impairment, as assessed by the ISI, had a higher risk of acute sleep impairment on the night before travel, such as poor sleep quality, difficulty falling asleep, and/or a sleep latency >30 min. In addition, the night before travel, a large majority of drivers reduced their sleep time and a third of them slept 5 h or less. In line with the study by Philip et al. who showed that sleep duration before departure is associated with the number of miles to be traveled (Philip et al., 1997), the present study further showed that the larger the delta between habitual and pre-travel TST, the more miles to be traveled. Hence, the longer the travel, the shorter the sleep time, and thus, the greater the decrease in sleep duration. Drivers also advanced their departure time as the number of miles to be traveled increased. This is of particular concern since lack of sleep has been related to decreased driving performances, when using driving simulations or real-life experiments, regardless of the perceived sleep sufficiency (Maia et al., 2013). These changes in sleep duration -which could lead to an increased sleep pressure- and in wake-up time -which could lead to a shift in the circadian rhythm of secretions- were transcribed in salivary cortisol concentrations. Consistent with studies that have reported elevated cortisol levels in healthy adults with insufficient sleep or longer wakefulness (Vgontzas et al., 2003; Guyon et al., 2014; Wright et al., 2015), cortisol levels increased with reduced sleep duration and in drivers who woke up earlier than usual on the day of departure. Given that drivers who participated in the study had been awake for more than 2 h, these results cannot be explained by a possible sampling during the cortisol awakening response, but it is questionable whether the afternoon cortisol quiescent period would be observed earlier due to earlier awakening.

The overall sleep characteristics studied herein were associated with daytime functioning. Chronic sleep disturbances, as assessed by the ISI, were associated with chronic and acute sleepiness. Acute sleep disturbances, as measured by poor sleep quality and difficulty falling asleep, were reported by about a fifth of the drivers, a large proportion of whom also suffered from acute sleepiness. Furthermore, these acute sleep disturbances were associated with high ESS scores, highlighting their chronicity beyond the day before departure. They were also associated with decreased attentional performances, as assessed by the BLAST. Poor sleep quality and sleep latency >30 min were associated with lower BLAST-stability and BLAST-intensity scores, reflecting attentional fluctuation. Poor sleep quality has been linked to a decreased activation in the prefrontal cortex (Telzer et al., 2013) and diminished connectivity in the Default Mode Network (DMN) at rest (Tashjian et al., 2018). A disturbance in the DMN function related to sleep quality could explain the poor BLAST performances, which involve the functional balance between the Dorsal Attention Network (DAN) and the DMN (Corbetta and Shulman, 2002; Petton et al., 2019). This suggests that acute sleep disturbances might be able to disrupt this equilibrium, promoting MLA, a few seconds during which cognitive resources are allocated involuntarily to task-unrelated processes (Weissman et al., 2006; Petton et al., 2019; Su et al., 2020). Leading to high variability in reaction time and response accuracy (Thieux et al., 2019), MLA could have a deleterious impact on driving ability (Su et al., 2020). While some sleep characteristics appeared to be associated with both sleepiness and BLAST scores, subjective sleepiness did not appear to be a good predictor of attentional performance as assessed by BLAST-stability and BLAST-intensity. It is important to note that the BLAST scores herein were mostly below normative values (Petton et al., 2019). This may be related to the assessment context and the level of motivation involved in the task, in addition to the sleep-related impact. This floor effect may make it difficult to establish a relationship between BLAST scores and sleepiness factors. However, the BLAST seems to be a promising tool to assess the quality of attentional stability on the road.

With regard to salivary markers, low sAA levels appeared to be more common in men, in people who snore, and in those with a sleep latency shorter than 30 min. High oxalate levels appeared to be more common in men and in smokers. Oxalate levels increased with chronic sleepiness as assessed by the ESS, but only in women. Thus, no sleep or acute sleepiness variable appeared to have a significant impact on sAA or oxalate levels. The first hypothesis to explain this lack of relationship is that sleepiness scales are not sensitive enough because of their limited correlations with objective sleepiness (Johns, 2000; Danker-Hopfe et al., 2001; Van Dongen et al., 2004; Short et al., 2010; Schreier et al., 2015) and because self-reported sleep measures have low reliability (O'Leary et al., 2015). Moreover, although sAA levels have been linked to changes in vigilance, no direct association with subjective sleepiness has been reported in the literature (Rabat et al., 2016; Pajcin et al., 2017).

Regarding sAA, contradictory results have been reported regarding its relation with sleep duration in lab studies as well as in studies without experimental sleep manipulation (Seugnet et al., 2006; Figueiro and Rea, 2011; Unno et al., 2013; O'Leary et al., 2015; Rabat et al., 2016; Pradhan and Sinha, 2018; Faraut et al., 2020; Gomez-merino et al., 2022). One hypothesis is that a single moderate night sleep restriction might not be sufficient to influence sAA the following day. Chronic (i.e., several weeks, sleep pathology) or intense sleep disruption (i.e., sleep deprivation or restriction protocols) might be required to observe a change (Van Lenten and Doane, 2016). The results regarding the effects of sleep quality are more consistent, showing that sleep fragmentation due to various sleep pathological conditions induced higher sAA levels, reflecting the SNS stimulation (Park et al., 2014; Ding et al., 2017; Yan et al., 2019). Herein, low sAA levels were more common among drivers who snore and those with a time to sleep latency shorter than 30 min. Although, in some, short sleep latency reflects good sleep quality, herein it is more likely to translate a higher homeostatic pressure, and thus, higher proportion of slow-wave sleep the night before travel, which seems related to a decrease in sAA the following day (Bachmann et al., 2012; Unno et al., 2017; Wei et al., 2017). Together with snoring, which produces higher sleep fragmentation, sAA levels appear to be lower in drivers with higher sleep pressure related to chronic disruption. Moreover, herein low sAA levels were more common in men than in women. This observed difference should be interpreted with caution since men also appeared to have higher sleep pressure (i.e., lower TST and more snoring) and earlier circadian rhythm (i.e., waking-up earlier) compared to women. Thus, this difference could reflect a sensitivity of sAA to sleep pressure (Rabat et al., 2016; Pajcin et al., 2017; Faraut et al., 2020) or be solely attributable to different sampling times during the circadian secretion cycle (Grant et al., 2019) between men and women. Indeed, sAA levels increase throughout the day with a nadir at the time of awakening (Chatterton et al., 1996; Nater et al., 2007; Rohleder and Nater, 2009), with a pattern opposite to cortisol levels (Nater et al., 2007). Even with this opposite nychthemeral variation, there was no correlation between sAA and cortisol levels, as already reported (Nater et al., 2007; Wolf et al., 2008). This is probably due to the difference in the release duration time between noradrenaline by the sympathetic nervous system and adrenal activation in the hypothalamo-pituitary axis (Danilenko et al., 2021; Pérez-Valdecantos et al., 2021).

However, the difficulty in showing differences in sAA levels according to sleep characteristics is probably due to the several factors that influence sAA levels. Of note, sAA was first evaluated as a marker of physiological and psychological stress (Chatterton et al., 1996; Granger et al., 2007; Nater et al., 2007). Although stress may produce an additive effect or alter the impact of sleep on salivary markers (Chatterton et al., 1996; Nater et al., 2007), it is questionable whether sleep disturbance can be equated with stress and therefore, whether sleep disruption can impact sAA independently of stress itself (LaVoy et al., 2020). Moreover, sAA levels can be modified by a large number of co-factors (Nater et al., 2007): those influencing the sympathetic system (e.g., stress, feelings, physical activity), those associated with saliva production (e.g., food, beverages), sampling method (i.e., active vs. passive) and location of collection (i.e., secretion from different glands) (Rohleder and Nater, 2009; Takeda et al., 2009), and other demographic factors (e.g., age, sex, BMI) (Granger et al., 2007; Nater et al., 2007; O'Leary et al., 2015). Indeed, the sAA variability has been reported before but remain largely unexplained. The number of copies of the sAA gene ranges from 1 to 15 between individuals, and positively influences the amount of protein, but this does not explain the full extent of the variability: two individuals with the same amount of protein can present very different levels of enzymatic activity and vice-versa (Mandel et al., 2010).

The relation between sAA levels and cognitive performances have also been investigated. During a sustained wake period (50 h), the variation of sAA levels was evaluated in 11 healthy adults. The best performances on the psychomotor vigilance test and driving simulation were obtained with the higher sAA levels, but only the first day (Pajcin et al., 2017, 2019b). These results are in agreement with those observed in the absence of sleep restriction, higher levels of sAA before a target detection task were associated with shorter reaction times at the first trial (Muehlhan et al., 2013). Similarly, other studies reported an association between decreased sAA levels and impaired performances (Rabat et al., 2016; Pajcin et al., 2019a; Faraut et al., 2020), which is not the case here although a high number of drivers performed poorly on the BLAST. Furthermore, these studies were conducted on small samples with highly selected subjects (i.e., mainly men, not older than 40 years, non-smokers). It is possible that in the present study, the relationship between sAA levels and performance is masked by noise intrinsic to the real-life conditions.

Although the literature on the topic is much less abundant for oxalate than sAA, it is possible that the various factors discussed above also influence salivary oxalate levels. Oxalate could reflect glucose metabolism (Gamelin et al., 2007; Weljie et al., 2015; Raap et al., 2018). Herein, high oxalate levels appeared to be more common in men and in smokers, two conditions with higher metabolic demands. Since smokers also had high ESS scores, oxalate might be sensitive to chronic sleepiness. Moreover, while oxalate levels were not associated with chronic sleepiness in men, they increased with higher ESS scores in women. These results are not consistent with original findings suggesting that oxalate levels are higher in women and decrease after sleep restriction (Kasidas and Rose, 1986; Wahl and Kallee, 1994; Weljie et al., 2015). However, studies are mostly conducted using blood samples, and results might differ when using saliva samples (Takeda et al., 2009; LaVoy et al., 2020).

To conclude, sAA and oxalate levels are influenced by many factors that must be considered to understand their fluctuations. Although sAA seemed to be a seducing biomarker for the assessment of sleepiness, the results of this study conducted in a heterogeneous population cannot conclude to its usefulness as an objective marker in the context of acute sleepiness. However, sAA and salivary oxalate might be useful as diagnostic tools to assess chronic perturbation of sleep and vigilance as it is the case in obstructive apnea syndrome or excessive daytime sleepiness (Grant et al., 2019), most efficiently as within-subject comparison and in tightly controlled conditions.

The present study has some limitations. First, the social desirability bias may have influenced the assessment of subjective sleepiness (Des Champs de Boishebert et al., 2021) and the strong association between chronic and acute sleepiness precludes distinguishing the effect of each independently on neurocognitive and biological markers. Then, drivers' sleepiness could have been influenced by non-controlled confounding factors such as undiagnosed or unreported sleep or mood disorders (e.g., sleep-disordered breathing, depression). Finally, a single time point for salivary samples, without a baseline condition, did not allow to assess intra-individual changes. Due to the high inter-individual variability in the baseline levels of sAA (Mandel et al., 2010), the changes in sAA due to sleepiness may have been smoothed. Also, the cross-sectional design of this study limits causation interpretation. Future studies could be conducted in a more homogeneous cohort controlled for chronic sleepiness, with several sampling time-points throughout the day, to better understand the fluctuation in salivary markers. As subjective sleepiness measures are not well suited to evaluate sleepiness repercussions on attention functioning, a future study might also use EEG-recording to assess the relationship between EEG-sleepiness measurements, salivary markers, and attentional performances.

## Conclusion

This study highlighted the relationship between sleep and sleepiness the next day, in a context of road-safety. Short TST was reported in more than one fourth of the drivers and, the higher the number of miles to travel, the shorter the sleep time. Poor sleep quality or difficulty falling asleep the night before travel were associated with increased sleepiness and decreased attentional abilities. Salivary markers were not found to be associated with acute sleepiness. Further studies are therefore needed to confirm their interests as objective markers to easily predict sleepiness, its repercussions, and the optimal timing of sleepiness countermeasures. Existing measures such as the SSS and the BLAST could be used by individual drivers to self-evaluate sleepiness.

## Data availability statement

The raw data supporting the conclusions of this article will be made available by the authors, without undue reservation.

## Ethics statement

The studies involving human participants were reviewed and approved by CPP Sud-Est II (ID-RCB 2016-A01511-50). Written informed consent for participation was not required for this study in accordance with the national legislation and the institutional requirements.

## Author contributions

AG, VH, LM, LS, and PF conceived and designed the study. MT, AG, VH, LM, LS, and PF acquired the data. MT and SP analyzed the data. MT, AG, VH, LM, J-PL, SP, LS, and PF interpreted the data and wrote the manuscript. All authors contributed to the article and approved the submitted version.

## Funding

This research was supported by the Vinci Autoroutes Foundation. MT was supported by the French Sleep Research and Medicine Society. The funders had no role in study design, data collection and analysis, decision to publish, or preparation of the manuscript.

## Conflict of interest

The authors declare that the research was conducted in the absence of any commercial or financial relationships that could be construed as a potential conflict of interest.

## Publisher's note

All claims expressed in this article are solely those of the authors and do not necessarily represent those of their affiliated organizations, or those of the publisher, the editors and the reviewers. Any product that may be evaluated in this article, or claim that may be made by its manufacturer, is not guaranteed or endorsed by the publisher.

## Supplementary material

Supplementary Figure S1 sAA, oxalate, and cortisol distributions before **(A1–C1)** and after **(A2–C2)** logarithmic (log) or square-root (sqrt) transformations.

Supplementary Figure S2 Directed acyclic graph model for salivary marker outcomes (salivary α-amylase and oxalate levels). In green, Sleepiness represents the explanatory variables (Epworth and Stanford total scores). In blue, Outcome represents variables to explain (herein, saliva outcomes based on the literature regarding α-amylase). In grey, collection time represents the adjusted variable. In white, all the other co-variables with a potential effect on Sleepiness and/or Outcome. BMI, Body Mass Index; ISI, Insomnia Severity Index; TST, Total Sleep Time.

Supplementary Figure S3 Co-variables correlation matrix with significant positive correlations in orange (+) and negative correlations in blue (-).

Supplementary Figure S4 Concentrations of salivary α-amylase (sAA) and cortisol according to time of inclusion and total awake time. Distribution of log-transformed sAA and cortisol concentrations according to the time of inclusion in hours **(A)** and to the total awake time in hours **(B)**. Each point represents one the concentration of sAA (blue) and cortisol (orange) for one subject.

Supplementary Figure S5 Chronic sleepiness measure according to oxalate levels. Each point represents the Epworth Sleepiness Scale (ESS) score for each driver according to the oxalate group based on a median-split categorization (blue: high, orange: low) and sex (left: women, right: men). The central line of boxplots corresponds to the median of each score, the upper and lower parts correspond to the first and third quartiles. There is no significant difference between groups.

Supplementary Table S1 Levels of salivary markers according to sex, tobacco consumption, age group, and sleep duration.

## Abbreviations

BLAST: Bron-Lyon Attention Stability Test
BMI: Body Mass Index
DAG: Directed Acyclic Graphic
ESS: Epworth Sleepiness Scale
ISI: Insomnia Severity Index
MLA: Momentary Lapses of Attention
MSLT: Multiple Sleep Latency Test
MWT: maintenance of wakefulness test
PVT: psychomotor vigilance task
sAA: salivary a-amylase
SEL: Socioeconomic Level
SSS: Stanford Sleepiness Scale
TST: Total Sleep Time.
