# Supplementary figures and images for: Interest of the BLAST paradigm and salivary markers for the evaluation of sleepiness in drivers
Source: Front Neurosci. 2022 Sep 7;16:991528. doi: 10.3389/fnins.2022.991528 (PMC9490274; doi:10.3389/fnins.2022.991528)

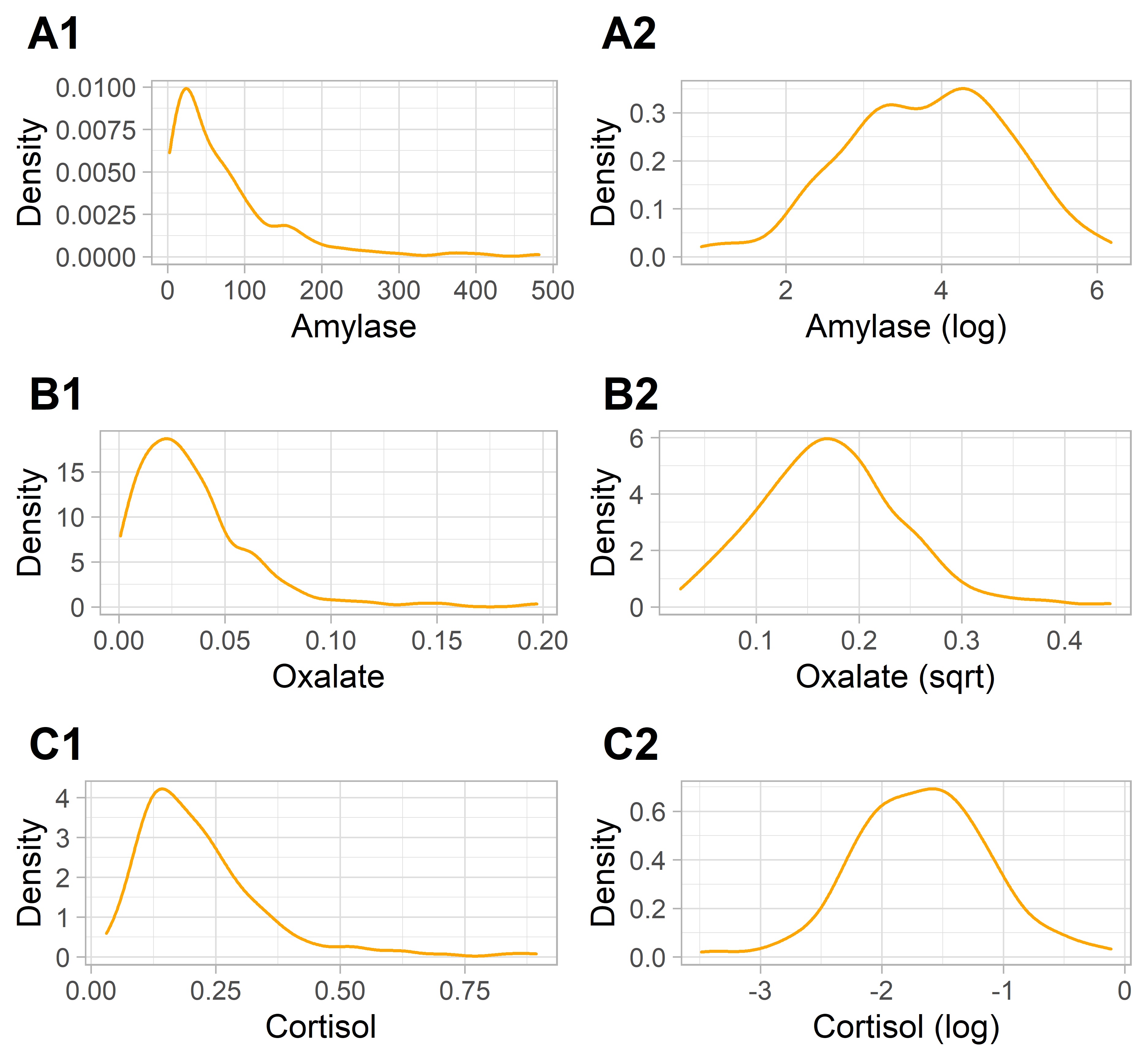

Supplement: Supplementary Figure S1 — sAA, oxalate, and cortisol distributions before (A1–C1) and after (A2–C2) logarithmic (log) or square-root (sqrt) transformations. [file Image_1.JPEG]

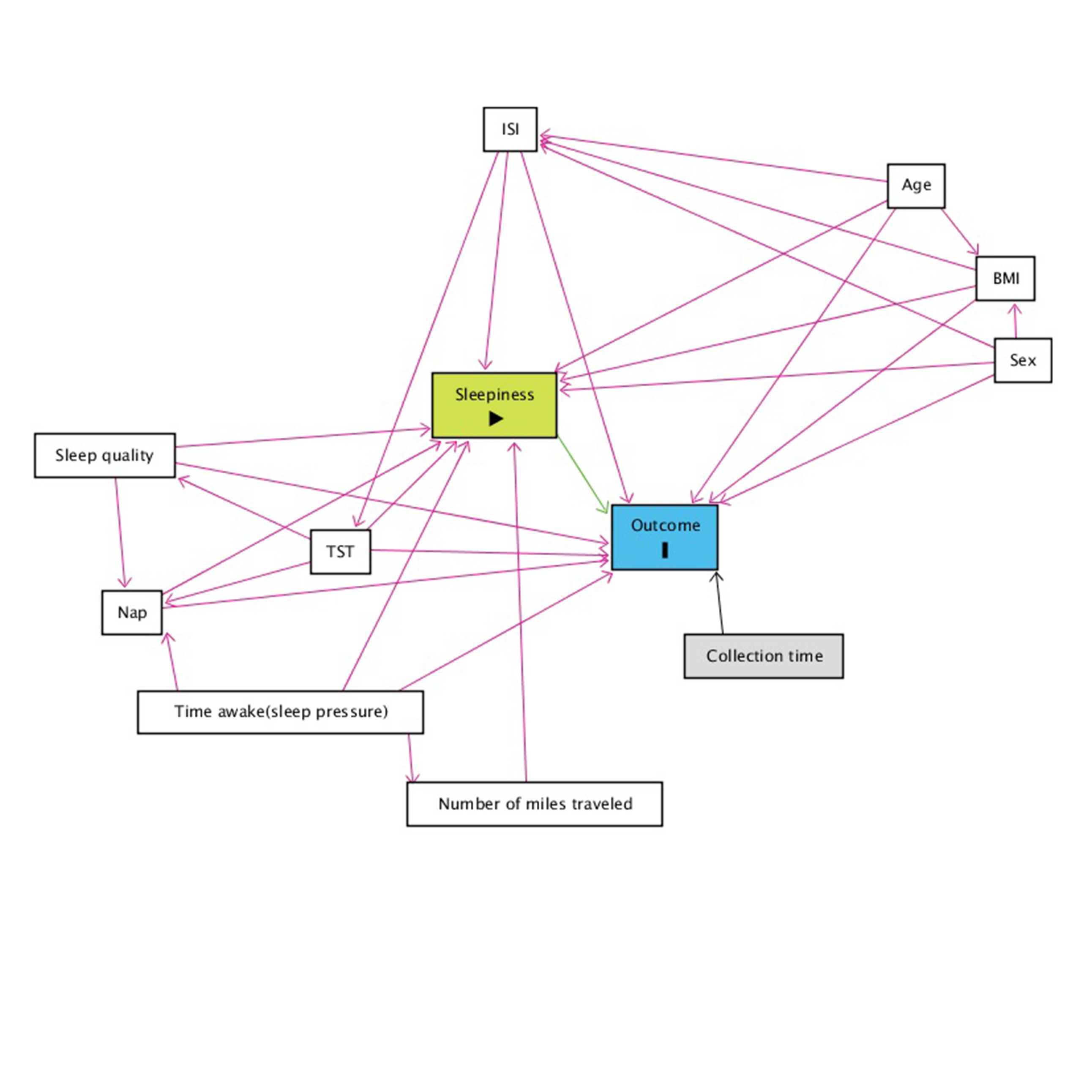

Supplement: Supplementary Figure S2 — Directed acyclic graph model for salivary marker outcomes (salivary α-amylase and oxalate levels). In green, Sleepiness represents the explanatory variables (Epworth and Stanford total scores). In blue, Outcome represents variables to explain (herein, saliva outcomes based on the literature regarding α-amylase). In grey, collection time represents the adjusted variable. In white, all the other co-variables with a potential effect on Sleepiness and/or Outcome. BMI, Body Mass Index; ISI, Insomnia Severity Index; TST, Total Sleep Time. [file Image_2.JPEG]

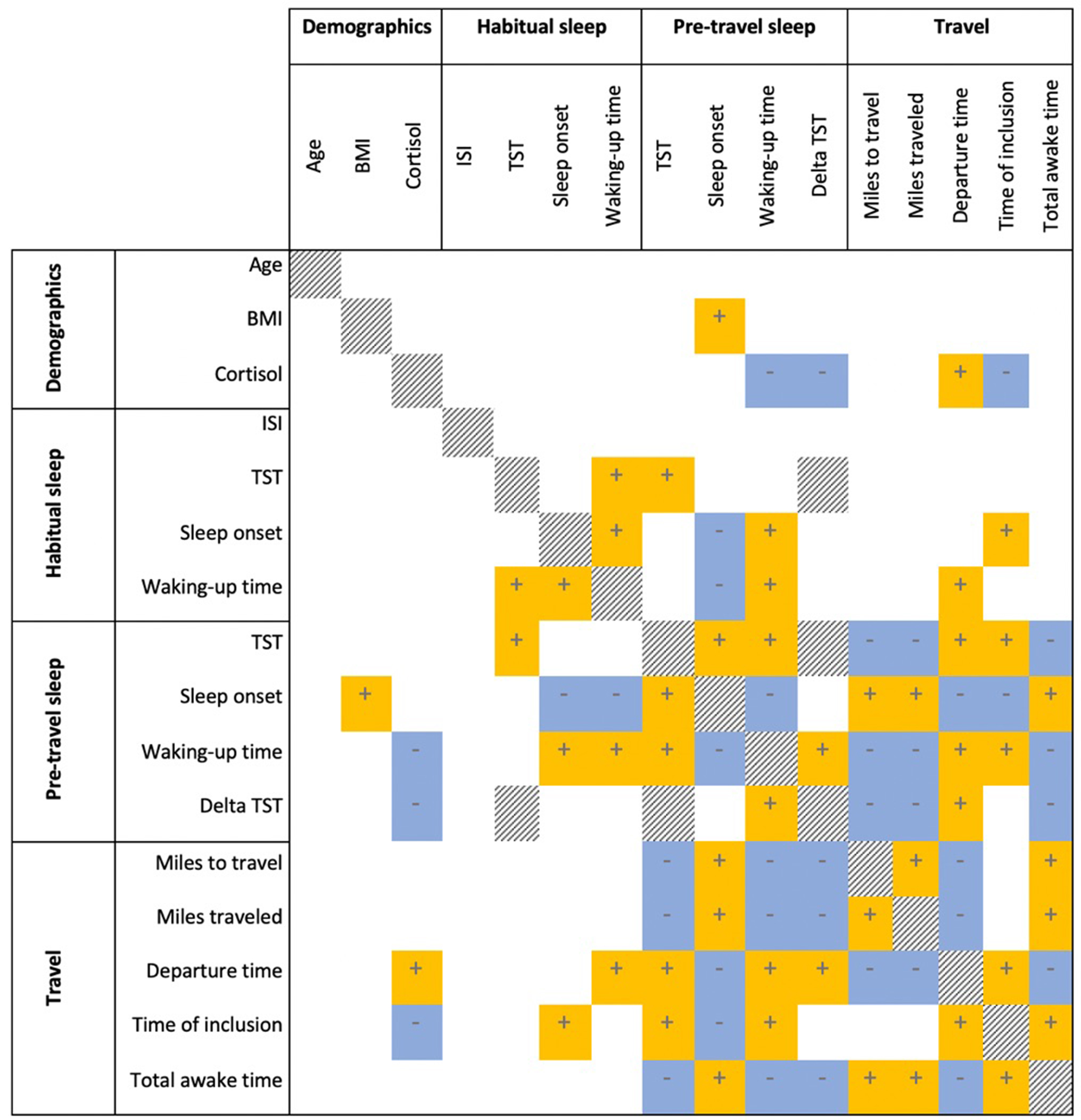

Supplement: Supplementary Figure S3 — Co-variables correlation matrix with significant positive correlations in orange (+) and negative correlations in blue (-). [file Image_3.JPEG]

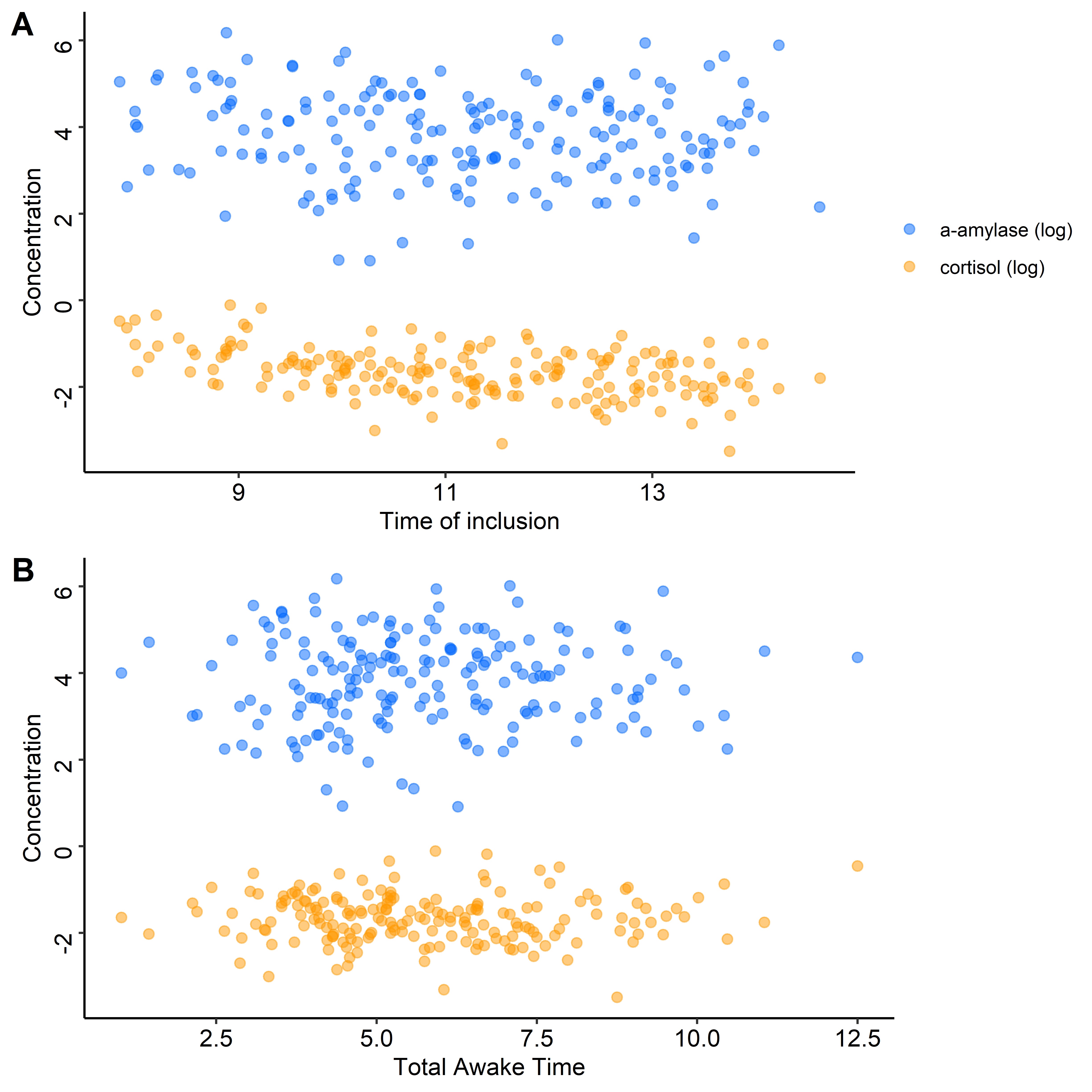

Supplement: Supplementary Figure S4 — Concentrations of salivary α-amylase (sAA) and cortisol according to time of inclusion and total awake time. Distribution of log-transformed sAA and cortisol concentrations according to the time of inclusion in hours (A) and to the total awake time in hours (B). Each point represents one the concentration of sAA (blue) and cortisol (orange) for one subject. [file Image_4.JPEG]

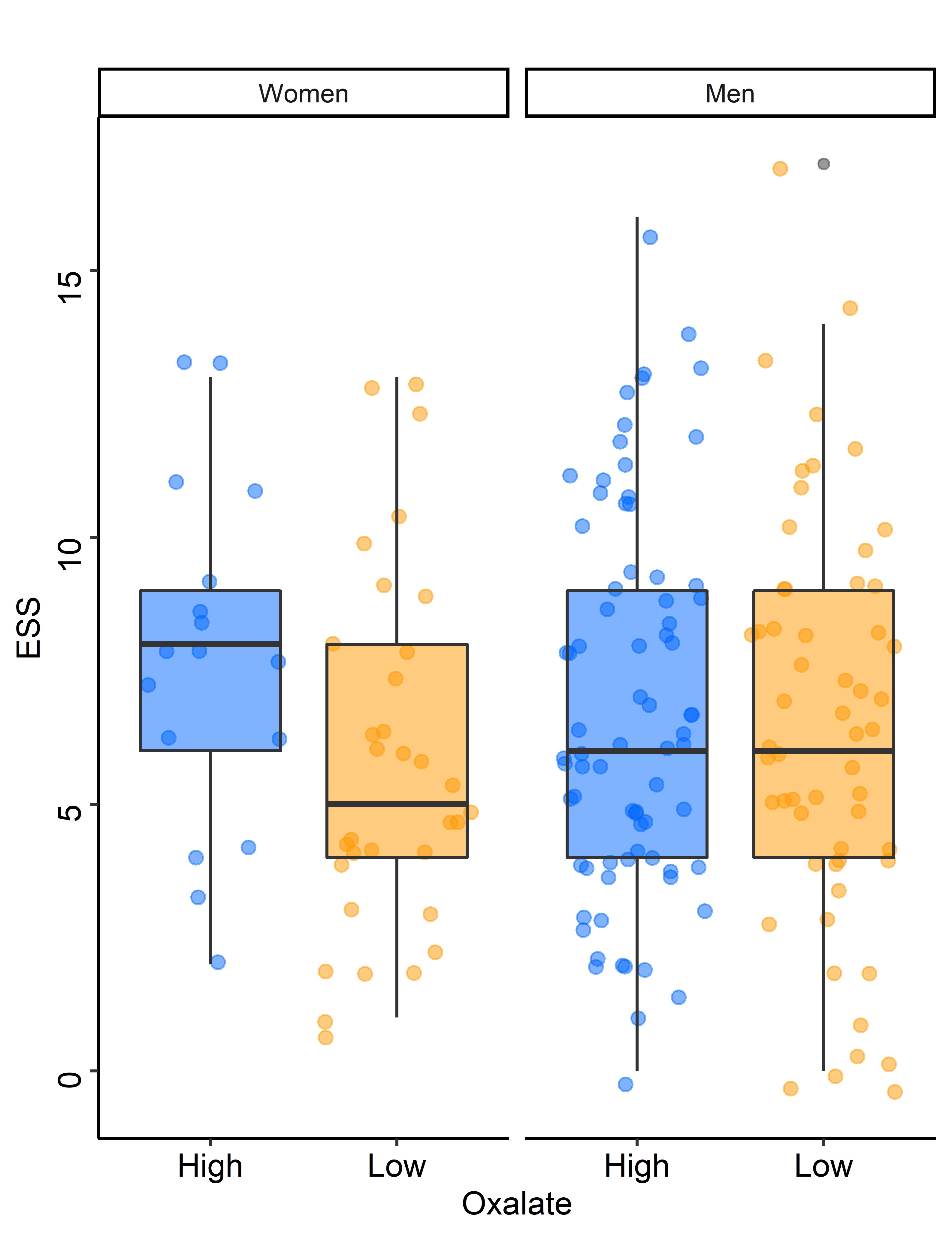

Supplement: Supplementary Figure S5 — Chronic sleepiness measure according to oxalate levels. Each point represents the Epworth Sleepiness Scale (ESS) score for each driver according to the oxalate group based on a median-split categorization (blue: high, orange: low) and sex (left: women, right: men). The central line of boxplots corresponds to the median of each score, the upper and lower parts correspond to the first and third quartiles. There is no significant difference between groups. [file Image_5.JPEG]
