# Supplementary material for: Interest of the BLAST paradigm and salivary markers for the evaluation of sleepiness in drivers
Source: Front Neurosci. 2022 Sep 7;16:991528. doi: 10.3389/fnins.2022.991528 (PMC9490274; doi:10.3389/fnins.2022.991528)
Supplement: Supplementary Table S1 — Levels of salivary markers according to sex, tobacco consumption, age group, and sleep duration. [file Table_1.DOCX]

Supplementary Material

# Supplementary Table

**Table S1.** Levels of salivary markers according to sex, tobacco consumption, age group, and sleep duration.

| **Sex** | **Men** | **N** | **Women** | **N** | **Total** | **N** |
| --- | --- | --- | --- | --- | --- | --- |
| α-amylase, U/ml, mean (SD) median (range) | 73.12 (80.38) 41.23 (2.49-482) | 135 | 76.01 (72.43) 57.83 (3.67-360.20) | 51 | 73.91 (78.10) 50.09 (2.49-482) | 186 |
| oxalate, U/ml, mean (SD) median (range) | 0.028 (0.025) 0.020 (0.002-0.112) | 135 | 0.038 (0.029) 0.031 (0.001-0.197) | 51 | 0.035 (0.028) 0.029 (0.001-0.197) | 186 |
| oxalate > 0.05, % (N) | 16% (8) | 135 | 22% (29) | 51 | 20% (37) | 186 |
| cortisol, µg/dL, mean (SD) median (range) | 0.224 (0.145) 0.193 (0.031-0.893) | 135 | 0.212 (0.105) 0.178 (0.063-0.575) | 51 | 0.221 (0.135) 0.191 (0.031-0.893) | 186 |
| **Tobacco** | **Smokers** |  | **Non-smokers** |  | **Total** |  |
| α-amylase, U/ml, mean (SD) median (range) | 71.48 (63.88) 54.87 (2.53-250.3) | 41 | 74.60 (81.86) 49.33 (2.49-482) | 145 | 73.91 (78.10) 50.09 (2.49-482) | 186 |
| oxalate, U/ml, mean (SD) median (range) | 0.050 (0.040) 0.040 (0.005-0.197) | 41 | 0.031 (0.023) 0.027 (0.001-0.121) | 145 | 0.035 (0.028) 0.029 (0.001-0.197) | 186 |
| oxalate > 0.05, % (N) | 34% (14) | 41 | 16% (23) | 145 | 20% (37) | 186 |
| cortisol, µg/dL, mean (SD) median (range) | 0.195 (0.077) 0.192 (0.070-0.386) | 41 | 0.228 (0.147) 0.185 (0.031-0.893) | 145 | 0.221 (0.135) 0.191 (0.031-0.893) | 186 |
| **Age group, years** | **(20, 47]** |  | **(47,74]** |  | **Total** |  |
| α-amylase, U/ml, mean (SD) median (range) | 71.66 (78.68) 46.99 (2.49-482) | 116 | 77.64 (77.55) 57.80 (2.53-380.7) | 70 | 73.91 (78.10) 50.09 (2.49-482) | 186 |
| oxalate, U/ml, mean (SD) median (range) | 0.035 (0.026) 0.028 (0.002-0.153) | 116 | 0.036 (0.032) 0.029 (0.001-0.197) | 70 | 0.035 (0.028) 0.029 (0.001-0.197) | 186 |
| oxalate > 0.05, % (N) | 22% (25) | 116 | 17% (12) | 70 | 20% (37) | 186 |
| cortisol, µg/dL, mean (SD) median (range) | 0.228 (0.140) 0.192 (0.031-0.833) | 116 | 0.209 (0.127) 0.177 (0.063-0.893) | 70 | 0.221 (0.135) 0.191 (0.031-0.893) | 186 |
| **Sleep duration** | **Short (< 400 min)** |  | **Normal (> 400 min)** |  | **Total** |  |
| α-amylase, U/ml, mean (SD) median (range) | 87.18 (91.98) 60.55 (2.49-409) | 46 | 74.62 (77.64) 51.3 (2.53-482) | 109 | 78.34 (82.05) 54.87 (2.49-482) | 155 |
| oxalate, U/ml, mean (SD) median (range) | 0.033 (0.023)  0.027 (0.001-0.089) | 46 | 0.037 (0.032) 0.030 (0.002-0.197) | 109 | 0.036 (0.029) 0.029 (0.001-0.197) | 155 |
| oxalate > 0.05, % (N) | 24% (11) | 46 | 22% (24) | 109 | 23% (35) | 155 |
| cortisol, µg/dL, mean (SD) median (range) | 0.218 (0.121) 0.184 (0.076-0.634) | 46 | 0.230 (0.135) 0.207 (0.031-0.893) | 109 | 0.226 (0.130) 0.196 (0.031-0.893) | 155 |
